# Mindfulness training for smokers via web-based video instruction with phone support: a prospective observational study

**DOI:** 10.1186/s12906-015-0618-3

**Published:** 2015-03-29

**Authors:** James M Davis, Alison R Manley, Simon B Goldberg, Kristin A Stankevitz, Stevens S Smith

**Affiliations:** University of Wisconsin School of Medicine and Public Health, Center for Tobacco Research and Intervention, Madison, USA; Department of Medicine, University of Wisconsin School of Medicine and Public Health, Madison, USA; Department of Counseling Psychology, University of Medicine, Wisconsin, Madison USA; Duke Center for Smoking Cessation, 2424 Erwin Road, Suite 201, Durham, NC 27705 USA; Department of Medicine, Duke University School of Medicine, Durham, USA

## Abstract

**Background:**

Many smokers are unable to access effective behavioral smoking cessation therapies due to location, financial limitations, schedule, transportation issues or other reasons. We report results from a prospective observational study in which a promising novel behavioral intervention, Mindfulness Training for Smokers was provided via web-based video instruction with telephone-based counseling support.

**Methods:**

Data were collected on 26 low socioeconomic status smokers. Participants were asked to watch eight video-based classes describing mindfulness skills and how to use these skills to overcome various core challenges in tobacco dependence. Participants received eight weekly phone calls from a smoking cessation coach who provided general support and answered questions about the videos. On the quit day, participants received two weeks of nicotine patches.

**Results:**

Participants were a mean of 40.5 years of age, smoked 16.31 cigarettes per day for 21.88 years, with a mean of 6.81 prior failed quit attempts. Participants completed a mean of 5.55 of 8 online video classes with a mean of 23.33 minutes per login, completed a mean of 3.19 of 8 phone coach calls, and reported a mean meditation practice time of 12.17 minutes per day. Smoking abstinence was defined as self-reported abstinence on a smoking calendar with biochemical confirmation via carbon monoxide breath-test under 7 parts per million. Intent-to-treat analysis demonstrated 7-day point prevalence smoking abstinence at 4 and 6-months post-quit of 23.1% and 15.4% respectively. Participants showed a significant pre- to post-intervention increase in mindfulness as measured by the Five-Factor Mindfulness Questionnaire, and a significant pre- to post-intervention decrease in the Anxiety Sub-scale of the Depression Anxiety and Stress Scale.

**Conclusions:**

Results suggest that Mindfulness Training for Smokers can be provided via web-based video instruction with phone support and yield reasonable participant engagement on intervention practices and that intervention efficacy and mechanism of effect deserve further study.

**Trial registration:**

ClinicalTrials.gov: NCT02164656, Registration Date June 13, 2014.

## Background

Tobacco use is the number one cause of preventable morbidity and mortality in the US [1], has devastating health effects worldwide [2], but is notoriously difficult to treat [3]. Approximately 50% of US smokers attempt smoking cessation each year [4], but discouragingly, abstinence rates remain less than 5% in unassisted attempts [5,6]. Since the advent of US public awareness of health risks from smoking [7], many smokers who have been able to quit by using medications or other available therapies have already quit, leaving a population of smokers today that is more dependent [8], and more resistant to available therapies [9]. Smokers now are most highly represented within low socioeconomic status (SES) populations [4,10] who often have limited access to effective therapies [11,12]. As the population of smokers becomes resistant to available therapies [13,14] there is a growing need for new, effective therapies that can be made widely available, especially to disadvantaged populations [15].

Currently the most widely accessible smoking cessation therapies are telephonic smoking cessation programs or “quit lines,” available to urban and rural smokers [16] in every US state [17]. Quit lines employ multiple therapeutic modalities including phone-based counseling, physician referral, mailed materials, subsidized pharmacotherapy [18], and web-based services [19,20]. Web-based services associated with quit lines may include written materials, short videos, interactive exercises, or access to online communities [21]. Web-based therapies have had a growing impact on low-SES smokers in the last decade, a change that is thought to be related to the development of wireless infrastructure in low SES regions [22], and adoption of smart phone use among disadvantaged populations [23,24].

A meta-analysis of US quit line therapies reported mean biochemically confirmed 6-month abstinence rates of 12.7% and 14-22% overall with use of subsidized medications [5,25,26]. A study on a quit line in England showed similar biochemically confirmed 6-month post-quit abstinence rates of 17.7% - 19.6% [27]. A 2013 Cochrane review [28] on telephone-based counseling in smokers, including only studies reporting biochemically confirmed abstinence, noted that variation in abstinence rates was associated with the use of medications and the number of proactive phone calls completed. Studies on quit lines that relied on self-report alone to measure abstinence (no biochemical confirmation), have reported substantially higher abstinence rates (e.g. 29.9% - 51.6%) [29-31].

Efforts to develop new and more effective therapies for smokers have led to the development and testing of “mindfulness training” for treatment of tobacco dependence. Mindfulness has been described as “paying attention in a particular way: on purpose, in the present moment, and nonjudgmentally” [32]. Put another way, mindfulness means bringing greater awareness to and acceptance of presently occurring thoughts, feelings, or physical sensations, ultimately allowing for a less reflexive and more thoughtful response to experiences as they arise [29,33]. Over the last seven years there has been a small but growing body of evidence supporting mindfulness training as a smoking cessation therapy [34] with studies showing that mindfulness training is associated with decreases in smoking urges [30], stress [33], anxiety [35-37], and depression [38], known predictors of smoking relapse [39-42].

Studies on mindfulness training for smokers include an initial pilot study in 2007 [43], a randomized trial favorably comparing mindfulness training to Freedom from Smoking, (American Lung Association cessation program) [44], a randomized trial favorably comparing mindfulness training to quit line [34], a study showing positive effects of mindful “urge surfing” [45], a study that showed favorable comparison between Acceptance Commitment Therapy (ACT) and Cognitive Behavioral Therapy [46], a study on with favorable comparison between ACT (provided in individual therapy) and control [47], and others [48,49]. Although these face-to-face therapies demonstrate considerable promise for smokers, their public health impact is ultimately limited to treatment of patients who live near providing treatment centers. Currently there is only one notable report on a web-based mindfulness intervention for smokers – namely a study conducted by Bricker et al. in 2013 [50]. This study provided a web-based video instruction on Acceptance Commitment Therapy (ACT) combined with proactive phone-counseling calls. ACT videos and phone calls integrated training in mindfulness with other techniques to help smokers quit. This intervention showed self-reported smoking abstinence of 22.8% at 3-months post-quit attempt, significantly higher than controls (10.3%) (*p* = 0.05) using Smokefree.gov, a widely used web-based intervention.

We report results of a pilot study on Mindfulness Training for Smokers Online (MTSO), a video-based intervention, developed for low SES populations cited above [44,51]. Up until now Mindfulness Training for Smokers (MTS) has only been used in face-to-face group interventions and has not been tested in a web-based video format with phone support. The primary objective of this study was to assess the feasibility of providing MTSO to smokers. Primary outcomes included completion of the five pre-quit video classes, the eight proactive phone calls, and the prescribed daily meditation and mindfulness practices. Also measured were changes in self-reported mindfulness, depression, anxiety and stress, and biochemically confirmed 4- and 24-weeks post-quit smoking abstinence.

## Methods

### Recruitment procedure

The study was funded through NIH/NIDA grant #K23DA022471, approved by the University of Wisconsin Health Science Institutional Review Board, and registered by Clinicaltrials.gov on 6/13/2014, protocol # NCT02164656. Participants were recruited over a 12-month period through a larger “parent” study targeted to low SES neighborhoods within a mid-sized city. The parent study employed phone screening followed by an orientation visit with additional screening and enrollment for those qualified and interested. Inclusion and exclusion criteria in the parent study required that participants be at least 18 years of age, smoke five or more cigarettes per day, use no other tobacco products, claim high motivation to quit, and consume no more than four alcoholic drinks on four or more days per week. Further details on the parent study are provided in Davis et al. 2014 [49]. If individuals were excluded from the parent study during phone or orientation screening specifically due to scheduling conflicts, they were called back later to assess their potential eligibility for participation in the MTSO study. During phone screening for the MTSO study, the only additional requirement for participation was self-reported access to the Internet. During a face-to-face MTSO study orientation session, study procedures were described including payment of $30 for attending each of the two face-to-face post-quit assessment visits (at 4 and 24 weeks post-quit attempt). If individuals decided to enroll in the study, they were provided with a user ID and password (written down for them), instructed in login procedures for the MTSO website, and asked to demonstrate the login procedure while at the study center. Potential participants were required to login to the MTSO website at home or at another off-site location to complete enrollment (a requirement employed to ensure that all study participants had Internet access).

### MTSO intervention

MTSO is a web-based smoking cessation intervention that provides instruction in mindfulness techniques through eight weekly video classes (five pre-quit video classes, a quit day class, and two post quit classes). Video classes provide instruction in skills such as mindfulness meditation, mindful walking, mindful eating, and smoking-specific skills such as mindfulness of smoking triggers, urges, emotions, and thoughts (see Table 1). The course also includes access to an online manual (109 pages) that includes information identical to that in the videos but with greater depth for participants who wanted to learn more than they could from the video alone. Use of the manual, however, was not required or stressed. Finally, participants were provided with web-based audio recordings of two guided meditation practices (15 and 30 minutes). Materials used were identical to those used in the face-to-face intervention from the parent study except that the video, manual, and audio recordings were provided only in a web-based format, not in physical form. Participants were instructed to watch one video per week, talk to a phone coach each week, practice meditation daily with the audio recording, and use other mindfulness practices spontaneously throughout the day. The Phone Coach was trained in procedures through the three-day MTS Teacher-Training Course (provided for the face-to-face MTS intervention), and had no additional training in addiction therapy. Phone calls were made to participants once per week on a specified day and the call for that week was not repeated if the participant did not answer. Phone counseling calls were a maximum of 15 minutes and structured around core concepts and practices for each of the eight video-based classes. Calls were not scripted, but the Quit Coach was instructed to address each core issue from the prospective class and to provide “listening only” for “non-core” issues that the participant might wish to discuss. Fidelity to call procedures was assessed by the study PI who listened to two phone calls per class and provided feedback on core issues. Phone calls were not recorded. After five weeks of weekly videos with calls, participants were asked to engage in a self-directed “Quit Day Retreat” and attempt smoking cessation. The recommended schedule of activities for the Quit Day Retreat included five hours of gentle mindfulness practices alternating with rest; these included mindful meditation, mindful walking, mindful eating, and mindful drawing or yoga. Each practice was scheduled to last 30 minutes, and participants were encouraged to modify the schedule to fit their needs. On the day of the Quit Day Retreat participants began a 2-week course of nicotine patches with dosing of 21 mg for > 10 cigarettes/day and 14 mg for ≤ 10 cigarettes/day. The phone coach made a call to participants on the Quit Day and made two weekly calls after that.

**Table 1:** **Mindfulness training for smokers via web-based video classes**

**Week**	**Class activity**
1	Mindfulness and mindfulness meditation
2	Mindful smoking and mindfulness of smoking triggers
3	Moments of mindfulness and mindfulness for emotions and stressful situations
4	Mindful walking and mindfulness for urges and withdrawal symptoms
5	Mindful eating and mindfulness for addictive thoughts
6	Quit Day Retreat (start two weeks of nicotine patches)
7	Mindfulness for relapse prevention
8	Long-term mindfulness practice

Note: The MTSO video classes provided training on how to use mindfulness to manage smoking relapse challenges. In addition, participants received access online to the MTSO Manual, Meditation CD and received weekly phone calls by an MTSO phone coach.

### Feasibility measures

The following measures were employed to assess intervention feasibility: **1)** phone call completion and length (recorded by the MTSO quit coach), **2)** video completion (reported on Course Evaluation - covered only 5 pre-quit videos), and also by Quit Coach via phone report **3)** website time (via a time-log function within the MTSO website), **4)** minutes of daily meditation (via meditation calendar with daily minutes meditated recorded from Video Class 1 until the 4-week post-quit study visit, and **5)** mindfulness practice (via Course Evaluation in which participants were asked to report the number of times per day that they engaged in various other mindfulness practices such as mindful walking, mindful eating, or moments of mindfulness).

### Self-report measures

In addition to these process measures, the following written surveys were obtained during baseline, 1-month post-quit, and 6-month post-quit assessment visits: **1)** Demographics Questionnaire, a non-standardized questionnaire including information on demographics, smoking history, and Internet use (baseline only), **2)** the Fagerstrom Test for Nicotine Dependence (FTND; only administered at baseline) [52], a six-item measure with internal consistency of α = .61 and correlation with biological indices of heaviness of smoking, **3)** the Five-Facet Mindfulness Questionnaire (FFMQ; all study visits), a 39-question survey with internal consistency between α = .75 and .91 [53-55] to assess mindfulness on five subscales (“observing,” “describing,” “acting with awareness,” “non-judging of inner experience,” and “non-reactivity to inner experience”). In addition to FFMQ subscale scores, a composite score was derived from the total of subscale scores, as in previous research [55], and **4)** the Depression Anxiety Stress Scales (DASS; all study visits), a 42-item measure with internal consistency of α = 0.96, 0.89 and 0.93 for depression, anxiety, and stress, respectively [56,57].

### Abstinence measures

Data on smoking status was collected at three assessment visits: baseline, 4- and 24-weeks post-quit attempt. The primary outcome was 7-day point-prevalence abstinence by Timeline Follow-Back smoking calendar (TLFB) at 4- and 24-weeks post-quit day confirmed by a carbon monoxide (CO) breath test [58]. Abstinence was defined as a CO monitor reading below 7 ppm (parts per million), a more stringent and contemporary standard used to minimize the possibility of false positive outcomes [59]. Determination of abstinence outcomes adhered to the intent-to-treat principle, such that failure to attend the 4- or 24-week assessment visits resulted in coding the participant as relapsed at that visit [60].

### Data analysis

Paired *t*-tests were used to examine changes in test scores over time and logistic regression was used to examine the effects of continuous predictors on abstinence (e.g., changes in self-report measures, practice time). All analyses were conducted using SPSS (Version 21). A sample size of 26 was determined to be cost effective for a pilot study designed primarily at obtaining feasibility data.

## Results

### Recruitment and demographics

**Figure 1:**
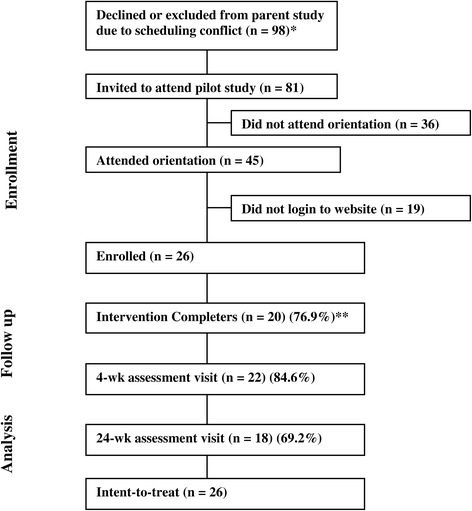
**Consort diagram.** *The parent study required that participants attend seven weekly face-to-face Wednesday evening meetings; 98 screened out of the parent study specifically for scheduling conflict. Of these 81 could be contacted by phone and were invited to the MTSO orientation. **Intervention Completion (n=20) was defined as self-report of making a quit attempt on the quit day.

Over a 12-month period, 98 individuals declined participation in the parent study due to scheduling conflicts. Of these 98 called, 81 were successfully reached by phone and invited to attend the MTSO orientation (Figure 1). All 81 pre-participants stated that they had Internet access (3 reported access through the public library) and all 81 were invited to the orientation. Of these, 45 attended the orientation, all signed consent, and all were given written instructions and a password to login to the MTSO website. Of these, 26 completed enrollment at their “home Internet site” by logging into the MTSO website. Enrolled participants had mean age of 40.5 years (*SD =* 38.48) and FTND = 3.92 (*SD* = 2.35). Most were Caucasian (88.5%), female (57.7%), and had education of high school or less (53.8%) reflecting efforts to recruit low SES population (Table 2). Post-hoc analyses demonstrated that there were no significant differences on any demographic variable between participants enrolled in MTSO study and the parent study. There was also no significant differences on any demographic variable between those who attended the orientation and did vs. did not enroll in the intervention (by home login).

**Table 2:** **Participant baseline characteristics**

GENDER	
Male	42.3%
Female	57.7%
RACE/ETHNICIY	
American Indian	0.0%
Asian	0.0%
African-American	3.8%
Caucasian	88.5%
Other	7.7%
Latino or Hispanic*	0.0%
EDUCATION	
Beyond high school	46.2%
High school or less	53.8%
Age	M = 40.50 (SD =13.48)
No. of cigarettes/day	M = 16.31 (SD = 9.06)
No. of years smoked	M = 21.88 (SD = 13.55)
No. of quit attempts	M = 6.81 (SD = 7.05)
FTND	M = 3.92 (SD = 2.35)

*Recruitment and intervention materials were in English only.

### Feasibility outcomes

The mean number of pre-quit online video classes completed by self-report was 4.64 of 5 (SD = 1.14, range = 0–5). By another method, Quit Coach report of online video classes attended, the mean was of 5.55 of 8 (SD = 2.48, range 0 – 8), with missing phone calls by Quit Coach coded as non-completion of the video classes. Mean logins to the MTSO website over the full intervention period was 8.05 (*SD =* 5.71, range = 0–25), with mean time per site login of 23.33 minutes (*SD =* 15.36) (recorded through the website). The mean number of proactive phone coach calls completed was 3.19 of 8 (*SD =* 3.06, range = 1–8). Mean reported meditation practice time was 12.17 minutes per day (*SD =* 8.15) (prescribed guided meditation recordings were 15 minutes/day for the first four weeks and 30 minutes/day until the 4-week study visit). Daily meditation time was not associated with abstinence (*p* = 0.71). Participants reported using informal mindfulness practices (e.g., moments of mindfulness, mindful walking, mindful eating, mindfulness of urges or triggers) an average of 5.0 times (*SD =* 6.15) per day up until the 4-week study visit. Intervention completion was defined as a self-reported quit attempt on the scheduled Quit Day. Using this definition “intervention completers” completed a minimum of 3 phone coach calls and 4 pre-quit video classes. Intervention completion rate was (20/26) 76.92% and was not associated with any demographic or baseline measure. Number of participants who used patches for the full two weeks was 12/26 (46.15%). There were no reportable medication reactions and patch use was not significantly associated with abstinence.

### Abstinence rates

Analysis of intent-to-treat samples showed biochemically confirmed 7-day point-prevalence smoking abstinence at 4- and 24-weeks post-quit attempt of 6/26 (23.08%) and 4/26 (15.38%), respectively. Other abstinence measures are provided in Table 3.

**Table 3:** **Abstinence rates at 4- and 24-weeks post-quit**

**Intent-to-treat analysis**	**(n = 26)**
4-week point prevalence	23.1%
24-week point-prevalence	15.4%
4-week continuous	15.4%
24-week continuous	7.7%
**Completer analysis**	**(n = 20)**
4-week point prevalence	30.0%
24-week point-prevalence	20.0%
4-week continuous	20.0%
24-week continuous	10.0%

**Intent-to-Treat Analysis:** Includes all subjects enrolled in the MTSO.

**Completer Analysis:** Includes only those subjects who self-reported a quit attempt on the quit day.

**Point Prevalence Abstinence:** CO < 7 ppm plus no cigarettes for last 7 days on TLFB.

**Continuous Abstinence:** CO < 7 ppm plus no cigarettes on TLFB plus statement of no cigarettes since the Quit Day.

Those who did not attend the assessment visit were recorded as smoking every day.

### Changes in mindfulness

Paired samples *t*-tests were used to measure change from baseline to 4- and 24-week post-quit study visits on measures of mindfulness (FFMQ; Table 4). Participants demonstrated predicted significant increases on FFMQ *observing* from baseline (*M =* 3.41, *SD =* .84) to the 4-week post-quit visit (*M =* 3.96, *SD =* .40), *t*(17) = −3.07, *p* = .007. This change persisted over time and was significant from baseline to the 24-week post-quit visit (*M =* 3.79, *SD =* .52), *t*(17) = −2.11, *p* = .051. Participants also showed predicted significant increases on FFMQ *non-judging* from baseline (*M* = 3.48, *SD* = .93) to the 24-week post-quit visit (*M* = 3.99, *SD* = .88), *t*(17) = −2.29, *p* = .035. Additionally, participants demonstrated significant increases on the FFMQ composite score from baseline (*M* = 3.39, *SD* = .58) to 4-weeks post-quit (*M* = 3.66, *SD* = .21), *t*(17) = −2.10, *p* = .051 and from baseline to the 24-weeks post-quit (*M* = 3.75, *SD* = .32), *t*(17) = −2.85, *p* = .011. There were no significant associations between FFMQ composite score or subscales and smoking abstinence.

**Table 4:** **Analysis of change in self-report measures over time**

**Measures (n = 18)**	***Baseline mean (SD)***	***4-weeks mean (SD)***	***24-weeks mean (SD)***	***t statistic, sig Baseline to 4-weeks***	***t statistic, sig Baseline to 24-weeks***
FFMQ^1^: Total score	3.39 (.58)	3.66 (.21)	3.75 (.32)	*t*(17) = −2.10 *p* = .05	*t*(17) = −2.85 *p* = .01*
FFMQ: Non-judging	3.48 (.93)	3.73 (.66)	3.99 (.88)	*t*(17) = −1.26 *p = .23*	*t*(17) = −2.29 *p* = .04*
FFMQ: Observing	3.41 (.84)	3.96 (.40)	3.79 (.52)	*t*(17) = −3.07 *p* = .01*	*t*(17) = −2.11 *p* = .05
FFMQ: Non- reactivity	3.12 (.72)	3.39 (.57)	3.43 (.54)	*t*(17) = −1.76 *p* = .10	*t*(17) = −1.95 *p* = .07
FFMQ: Describing	3.65 (.68)	3.68 (.58)	3.89 (.61)	*t*(17) = −.23 *p* = .82	*t*(17) = −1.71 *p* = .11
FFMQ: Acting with awareness	3.27 (.88)	3.49 (.68)	3.62 (.48)	*t*(17) = −1.26 *p* = .23	*t*(17) = −1.81 *p* = .09
DASS^2^: Total score	0.66 (.60)	0.50 (.27)	0.39 (.34)	*t*(17) = 1.24 *p* = .23	*t*(17) = 1.75 *p* = .10
DASS: Depression	0.49 (.69)	0.33 (.30)	0.25 (.28)	*t*(17) = .92 *p* = .37	*t*(17) = 1.33 *p* = .20
DASS: Anxiety	0.49 (.50)	0.31 (.32)	0.28 (.32)	*t*(17) = 2.12 *p* = .05*	*t*(17) = 1.71 *p* = .11
DASS: Stress	1.02 (.75)	0.85 (.54)	0.65 (.56)	*t*(17) = .84 *p* = .41	*t*(17) = 1.97 *p* = .07

^1^FFMQ = Five Facet Mindfulness Questionnaire; ^2^DASS = Depression Anxiety Stress Scales.

^*^Values with asterisk are statistically significant (*p < .05).

### Changes in depression anxiety and stress

Scores on the DASS demonstrated predicted significant decreases on the anxiety subscale from baseline (*M =* .49, *SD =* .50) to 4-week post-quit visit (*M =* .31, *SD =* .32), *t*(17) = 2.12, *p* = .049. Depression and stress subscales demonstrated change in the predicted direction (decrease over the intervention period), but changes were non-significant. There were no significant associations between DASS composite score or subscales smoking abstinence.

## Discussion

### Feasibility - intervention compliance

Compliance with the MTSO intervention was relatively good, with participants from low SES neighborhoods showing they were able to use the website, complete phone calls with the Phone Coach, and comply relatively well with daily meditation and mindfulness practices. Completion of web-based course material was reassuring with participants reporting completion of 4.64 of 5 pre-quit videos, and the internal website log registering a mean of 8.05 total logins with 23.33 minutes per login. The mean number of calls completed with the Phone Coach was a bit lower at 3.19 of 8 possible calls. One reason phone call completion may have been somewhat low is that phone calls were made only one day per week and repeated attempts were not made to contact participants who missed calls. Research on quit lines suggests that there is a correlation between number of calls completed and abstinence rates [61], and that on average, smokers complete roughly half of the number of calls recommended [27].

### Feasibility - medication use

There are a couple of reasons that may have lead to a lack of association between medication use and abstinence. The first is that while the behavioral treatment employed was quite intensive (mean completion of 4.64 of 5 pre-quit videos and 3.19 phone calls), the pharmacotherapy employed was relatively non-intensive – two weeks of nicotine patches – which though effective is less so than longer therapy (e.g., 12 weeks) [5]. It is also likely that the study was underpowered to demonstrate differences between compliant (*n* = 12) and non-compliant patch users (*n* = 14).

### Feasibility - practice time

Adherence to daily meditation was modest but acceptable, with a mean of 12.17 minutes meditation practice per day. This is a lower rate of daily meditation practice than was found in Mindfulness Training for Smokers in a face-to-face format (21.6 minutes per day) [44]. Participants reported using other mindfulness practices (e.g., mindful walking, mindful eating, mindfulness of urges) a mean of 5.0 (SD 6.15) times per day, suggesting that mindfulness practices were taught at least somewhat successfully through web-based instructional videos with phone support. The lack of association between meditation time and smoking cessation outcomes has been seen in other studies using mindfulness training for smokers [43,49], and new data is now emerging to suggest that practice quality (self-reported on a practice quality measure) may be a better predictor than practice time of at least some psychiatric outcomes [62].

### Acquisition of mindfulness

An important question to address when testing mindfulness training in a new format is whether participants are able to acquire mindfulness skills through this format. The most accepted secular training in the US is Mindfulness-Based Stress Reduction (MBSR) [30], which provides mindfulness training in face-to-face group format. This study suggests that participants did in fact acquire mindfulness skills as reflected in significant increases in pre- to post-intervention FFMQ subscales “observing” and “non-judging,” and FFMQ composite scores. The increases in FFMQ subscales and composite provides support to the notion [34] that acquisition of mindfulness skills does not require face-to-face instruction, but might be successfully taught via web-based video instruction with phone support. It should be noted that the FFMQ has limitations typical of any self-report measure including susceptibility to social desirability bias [63] or “halo” effects [64]. To better assess mindfulness acquisition within this format, and reduce such potential bias, it would be helpful to compare MTSO to an active control in a randomized study design. The finding that FFMQ scores were not associated with abstinence may have been because mindfulness skills were not affecting smoking behavior, or may been due to insufficient power to detect this effect in a small study.

### Changes in anxiety, depression and stress

Participants showed significant decreases in anxiety relative to baseline, as measured by scores on the DASS, which was promising given that research has implicated anxiety as a major cause of smoking relapse [65]. Participants also showed a drop in pre- to post-intervention depression and stress subscales and DASS composite score, but these changes were non-significant. A study by Goldberg et al. (2014) on Mindfulness Training for Smokers (in face-to-face group format) [66] showed decrease in self-reported pre- to post intervention DASS stress subscale and hair cortisol, suggesting that MTS in a face-to-face format does in fact lead to decreased stress. Statistically non-significant decreases in stress scores on DASS in this study suggests that MTSO has less robust effect on participants than MTS in a face-to-face format, or that the study was insufficiently powered to demonstrate a significant change. The finding that DASS scores were not associated with abstinence may have been because changes in depression, anxiety or stress were not affecting smoking behavior, or may been due to insufficient power.

### Abstinence outcomes

Biochemically confirmed abstinence rates of 23.1% at 1-month and 15.7% at 6-month post-quit are on par with web-based quit line interventions cited in a recent meta-analysis [5], and even perhaps encouraging when targeting recruitment to a low SES population. Abstinence rates are similar to those found by Bricker et al. [50] who report a 3-month post-quit 30 day continuous abstinence rate of 22.8% (not-biochemically confirmed). Bricker’s ACT intervention was similar in a number of ways; it was an 8-part program using web-based video instruction, and phone-support and provided training in a mindful approach to urges, emotions and thoughts.

### Limitations

The major limitations of this feasibility study were it’s small sample size and lack of a control group. In a pilot study such as this, findings on abstinence rates and self-report measures can at most suggest the possibility of a therapeutic effect. Another potential confound in this study is that participants were recruited from a pool of individuals who reported scheduling conflicts. It is plausible that those who report scheduling conflicts were different in some respects from the smoking population as a whole, thus limiting the generalizability of results. For example, patients with busy schedules may be more active, have more demanding jobs and be more motivated to quit smoking. Additionally, the requirement of participants to have Internet access may have contributed to selection bias, perhaps selecting participants who were more advantaged and thus more likely to be compliant and maintain abstinence. The finding that there were no significant demographic differences between MTSO and parent study participants suggests that employment of inclusion criteria of scheduling conflicts and Internet access appear to have had only modest impact on sample selection. The fact that there were no demographic differences between those who came to the orientation and enrolled vs. did not enroll suggests that the requirement to login at home did not meaningfully impact sample selection. That being said, if larger sample were studied, significant differences between these groups may have been found.

## Conclusions

This study evaluated a novel intervention that uses web-based video instruction and phone counseling to teach mindfulness skills to smokers. Such an intervention has the potential for large-scale web-based dissemination and could conceivably be used as a complementary therapy by a tobacco quit line. If MTSO were used along with other quit line treatments, it could potentially lead to higher abstinence rates in quit line callers. Because MTSO functions as a video-based intervention, it might conceivably be provided to a wide population at minimal expense. Even if only a modest portion of the smoking population were responsive to web-based mindfulness training, it could have a meaningful public health impact due to the wide accessibility of the Internet. Given the promising feasibility findings in this study, but considerable limitations due to small sample size and lack of control, we suggest that web-based mindfulness training for smokers would appear to merit further research.
